# Integrated Liver Multi-Omics Reveals Beneficial Effects and Potential Mechanisms of Noni Fruit Flavonoids on Antioxidant Status and Anti-Inflammatory Responses in Cashmere Goats

**DOI:** 10.3390/antiox15080987

**Published:** 2026-08-09

**Authors:** Shuhui Dong, Qingyue Zhang, Hao Yu, Yanli Zhao, Yu Xin, Yongmei Guo, Xiaoyu Guo, Yuanqing Xu, Sumei Yan

**Affiliations:** 1 Key Laboratory of Animal Nutrition and Feed Science of Inner Mongolia Autonomous Region, College of Animal Science, Inner Mongolia Agricultural University, Hohhot 010018, China; shuhuidong@emails.imau.edu.cn (S.D.); zhangqy2024@imau.edu.cn (Q.Z.); haoyu@emails.imau.edu.cn (H.Y.); ymguo2020@imau.edu.cn (Y.G.); gxy2024@imau.edu.cn (X.G.); xuyuanqing@imau.edu.cn (Y.X.); 2 National Technology Innovation Center for Prataculture, Hohhot 010010, China; yuxin119822@163.com

**Keywords:** noni fruit, flavonoids, cashmere goat, antioxidant, anti-inflammatory

## Abstract

This study investigated the effects of noni fruit flavonoids (NFF) on antioxidant status and anti-inflammatory responses in cashmere goats and explored the underlying mechanisms based on liver transcriptomics and metabolomics. Sixteen male Albas cashmere goats were randomly assigned to two groups: a control (CON) group and a group supplemented with 0.1% NFF. The adaptation and trial periods were 2 and 12 weeks, respectively. The results showed that NFF significantly enhanced antioxidant capacity, with mean increases of 32.9%, 20.7%, and 35.5% for total antioxidant capacity (T-AOC), glutathione peroxidase (GPx), and catalase (CAT) activities, respectively, across serum, liver, spleen, and thymus (*p* < 0.05). NFF also modulated the inflammatory profile, with a mean increase of 18.0% for interleukin (IL)-10 and mean reductions of 18.6%, 19.4%, and 16.7% for IL-1β, IL-2, and tumor necrosis factor (TNF)-α, respectively, across the serum, liver, and thymus (*p* < 0.05). Integrative transcriptomic and metabolomic analyses suggested that the AMPK signaling pathway, glutathione metabolism, and TGF-β signaling pathway may be critically involved in mediating these effects. *GPX3*, *GPX8*, *GCLM*, *NFKB1*, and *IL1B* were identified as key candidate genes, while adenosine-5′-monophosphate (AMP), cysteinylglycine, and pyroglutamic acid were key differential metabolites. Dietary supplementation with 0.1% NFF enhanced the antioxidant levels and anti-inflammatory response in cashmere goats.

## 1. Introduction

Albas cashmere goats, a dual-purpose breed valued for both fiber and meat, inhabit the semi-desert grassland of western Inner Mongolia, China. Their meat commands a premium in local markets for its high protein, low fat, and abundant iron and amino acids, and notably mild flavor without the typical mutton odor. However, in recent decades, the simultaneous degradation of natural grasslands and rising consumer demand for meat have driven a shift toward intensive, high-concentrate feeding systems [1,2]. While this intensification boosts production efficiency, it frequently induces ruminal acidosis and decreased meat quality. Low-concentrate confined feeding circumvents these issues, promotes rumen development, improves meat fatty acid profiles, and lowers feed costs. Yet, the high stocking densities inherent to this system still provoke oxidative stress and immunosuppression, predisposing animals to metabolic diseases and chronic inflammation that constrain development of the goat industry [3]. Improving the antioxidant status and anti-inflammatory capacity in cashmere goats under these conditions is imperative.

Flavonoids, a class of plant secondary metabolites, are well-established for their antioxidant and anti-inflammatory bioactivities. For instance, mulberry leaf flavonoids exhibit excellent free radical-scavenging capabilities [4], and studies in ruminants have shown that naringin supplementation enhances antioxidant enzyme activity, reduces lipid peroxidation, and regulates the levels of immune factors [5]. Noni fruit (*Morinda citrifolia*), prevalent in Hainan and other regions of China, contains over 200 bioactive compounds and is particularly rich in flavonoids. These phytochemicals underpin its diverse pharmacological properties, such as antioxidant, anti-inflammatory, hypoglycemic, and hypolipidemic activities [6,7]. Capitalizing on these properties, our group previously extracted noni fruit flavonoids (NFF) and demonstrated their robust free radical-scavenging capacity against 1,1-diphenyl-2-trinitrophenylhydrazine (DPPH) and 2,2-diazo-bis (3-ethyl-benzothiazol-6-sulfonic acid) diammonium salt (ABTS). Furthermore, it had a strong reducing power for Fe^3+^ [8]. Subsequent in vitro rumen fermentation experiments identified 0.1% NFF as the optimal dose for improving rumen fermentation and mitigating oxidative stress in cashmere goats on high-concentrate diets. Glutathione peroxidase (GPx) activity was enhanced by 0.1% NFF, while levels of malondialdehyde (MDA), lipopolysaccharide (LPS), and histamine (HIS) were decreased [9,10]. Parallel benefits were observed in low-concentration fermentation models supplemented with the same dose. At present, research on NFF remains largely confined to extraction methodologies and in vitro bioactivity screening; its in vivo antioxidant and anti-inflammatory effects in ruminants remain virtually unexplored.

To bridge this gap, we integrated serum and tissue antioxidant and anti-inflammatory profiling with liver transcriptomic and metabolomic analyses to elucidate the regulatory mechanisms underlying 0.1% NFF in cashmere goats. This multidimensional framework enabled simultaneous assessment of phenotypic, transcriptional, and metabolic changes, providing complementary mechanistic insights. The findings are expected to advance strategies for sustainable cashmere goat production, facilitate value-added utilization of noni fruit resources, and support the development of novel green feed additives.

## 2. Materials and Methods

### 2.1. Preparation of NFF

The air-dried slices of noni fruit were obtained from the noni fruit cultivation base in Wuzhishan City, Hainan Province. The slices were dried at 65 °C, crushed into powder, and sieved through a 40-mesh sieve to obtain a uniform noni fruit powder. NFF (yield: 27.1%) was extracted via an ultrasonic-assisted method at the Ruminant Nutrition and Feed Development Laboratory. The extract is a crude preparation rather than a purified isolate, with a total flavonoid content of 23.14 mg rutin equivalents/g dry matter. UPLC–MS/MS (Shimadzu CBM30A/UFLC system, Kyoto, Japan; 6500 Q TRAP, AB Sciex Pte. Ltd., Shanghai, China) profiling identified flavonoids as one of the major constituent classes, accounting for 9.24% of the total ion intensity, with rutin, quercetin, and kaempferol as the most abundant flavonoid monomers [10,11].

### 2.2. Animals, Experimental Design, and Diets

All animal experimental procedures were approved by the Animal Ethics and Welfare Committee of Inner Mongolia Agricultural University and were conducted in accordance with the regulations of the Laboratory Animal Science and Technology Committee of the Standardization Administration of China. All procedures complied with the national guidelines for ethical review and animal welfare.

This research employed a completely randomized design with a single factor. Sixteen male Arbas cashmere goats were selected and randomly assigned to either a control group (CON), which received a basal diet, or an NFF group, which was given a basal diet supplemented with 0.1% NFF. The study included a 2-week adaptation phase followed by a 12-week formal trial. The early experimental phase spanned weeks 1 to 6, while the late experimental phase covered weeks 7 to 12. The basal diet was prepared in line with the nutritional requirements for meat goats (NY/T816-2021) [12], maintaining a concentrate-to-roughage ratio of 20:80. The composition and nutrient content of the diets are detailed in Table 1.

### 2.3. Sample Collection and Determination of Antioxidant and Inflammatory Parameters

During the 6th and 12th weeks of the experiment, blood samples from the jugular vein were taken from all goats both in the morning before feeding and three hours post-feeding. These samples underwent centrifugation at 3500 r/m for 15 min, after which the serum was extracted and stored at −20 °C. Upon completion of the trial, all cashmere goats were slaughtered, and their liver, spleen, and thymus were kept at −80 °C.

The concentrations of GPx, T-SOD, catalase (CAT), total antioxidant capacity (T-AOC), and MDA in the serum, liver, spleen, and thymus were measured using commercial kits from Nanjing Jiancheng Bioengineering Institute, Nanjing, China. The levels of IL-1β, IL-2, IL-6, IL-10, TNF-α, inducible nitric oxide synthase (iNOS), HIS, and LPS in these tissues and serum were assessed using commercial enzyme-linked immunosorbent assay (ELISA) kits (Quanzhou Ruixin Biotechnology Co., Ltd., Quanzhou, China), following the manufacturer’s protocol.

### 2.4. Liver Transcriptome Sequencing and Enrichment Analysis

For transcriptomic analysis, seven liver samples from each group were initially selected for sequencing. However, one sample in the NFF group was damaged due to improper storage and was excluded from further analysis. Consequently, the final sample sizes were *n* = 7 for the CON group and *n* = 6 for the NFF group. Differential expression analysis was performed using DESeq2, which accommodates unequal group sizes.

Total RNA was isolated from the samples using the TRIzol Kit (Takara, Dalian, China). The DNase I Kit (Takara) was employed to eliminate genomic DNA. Subsequently, the concentration and purity of the extracted RNA were assessed with a Nanodrop 2000 spectrophotometer (Thermo Fisher Scientific, Wilmington, DE, USA), while agarose gel electrophoresis was utilized to evaluate RNA integrity, and the RIN value was measured using Agilent 5300 Bioanalyzer (Agilent Technologies, Santa Clara, CA, USA). RNA samples meeting the quality criteria of total RNA ≥ 1 ug, RNA concentration ≥ 30 ng/μL, RIN ≥ 6.5, OD260/230 ≥ 1.0, and OD260/280 ≥ 1.8 were chosen for constructing the sequence library. Quantification was carried out with Qubit 4.0 fluorometer (Thermo Fisher Scientific, Waltham, MA, USA), and samples were mixed based on data proportions before undergoing Bridge PCR amplification. Sequencing was conducted on the Illumina NovaSeq 6000 platform (Illumina, San Diego, CA, USA). The sequencing tasks were executed by Shanghai Majorbio Biomedical Technology Co., Ltd., Shanghai, China. To quantitatively assess gene expression levels and identify differentially expressed genes (DEGs) among samples, RSEM (v1.3.3) and DESeq2 software (v1.42.0) were employed, with significant DEGs being screened using *p*-value < 0.05 and |log_2_FC| ≥ log_2_(1.2) as criteria. Consequently, pathway-level KEGG enrichment analysis of the identified DEGs was performed using Python SciPy software (v1.10.1).

### 2.5. Quantitative Real-Time Polymerase Chain Reaction (qRT-PCR)

Reverse transcription of liver total RNA was performed using the Haifai II First Strand cDNA Synthesis SuperMix Kit (Yisheng Biotechnology Co., Ltd., Shanghai, China). To confirm the sequencing results’ accuracy, 10 DEGs, including *GPX3*, *GPX8*, *NFKB1*, *IL1B*, *GCLM*, *CASP3*, *PPARGC1A*, *PRKAA2*, *NLRP12*, and *ACVR1C*, were chosen from the liver DEGs for qRT-PCR analysis. *β-actin* and *B*_2_*M* were used as internal reference genes [13]. Specific primers for the target genes were designed using Primer 5.0 software, and the primer sequences are detailed in Table S1. The LightCycler 480 Real-Time PCR System (Roche Diagnostics, Basel, Switzerland) was used for qRT-PCR analysis. Cycling conditions were as follows: predenaturation at 95 °C for 300 s; 40 cycles of 95 °C for 10 s, 55–60 °C for 20 s, and 72 °C for 20 s. Relative gene expression was calculated using the 2^−ΔΔCT^ method [14].

### 2.6. Liver Untargeted Liquid Chromatography–MS Metabolomic Analysis

Seven biological replicates were selected from each group for metabolomic analysis. Liver tissue (50 mg) was extracted using a pre-chilled methanol/acetonitrile/water mixture (2:2:1, *v*/*v*/*v*) via low-temperature ultrasonication. After incubation at −20 °C for 30 min and centrifugation at 15,000× *g* for 15 min at 4 °C to remove proteins and impurities, the supernatant was injected for analysis. Utilizing liquid chromatography–mass spectrometry (LC-MS) technology, which includes an ACQUITY UPLC HSS T3 C18 column (100 mm × 2.1 mm, 1.8 μm; 40 °C; 0.4 mL/min; Waters Corporation, Milford, MA, USA) and a Thermo Q Exactive HF-X high-resolution mass spectrometer (Thermo Fisher Scientific, Waltham, MA, USA), data was comprehensively collected in both positive (+3.5 kV) and negative (−2.8 kV) ion modes across the m/z range 70–1050, ensuring that the mass error remained within 5 ppm. A pooled QC sample was injected every 8 consecutive runs to monitor system stability. Raw data were processed using XCMS (v3.20.0) and MS-DIAL (v4.90). Metabolite identification was based on MS/MS spectral matching against the HMDB, Metlin, and Majorbio databases (MSI Level 2). Multivariate analysis was performed using PCA and OPLS-DA. Differential metabolites were screened based on VIP > 1.0 (from OPLS-DA) and *p*-value < 0.05. Given the robust multivariate filtering applied, correction for multiple comparisons (FDR) was omitted to avoid excluding biologically relevant signals. Pathway enrichment was performed using the KEGG database via the Majorbio Cloud Platform. The workflow was completed by Shanghai Majorbio Biomedical Technology Co., Ltd.

### 2.7. Statistical Analysis

The data on antioxidant and immune factors were analyzed using SAS 9.4 software. Normality was assessed using the Shapiro–Wilk test. Student’s *t*-test was used for data with normal distribution, and the Wilcoxon rank-sum test was used for data without normal distribution. A *p*-value < 0.05 was considered indicative of a statistically significant difference between groups, while 0.05 ≤ *p* < 0.10 was considered a tendency towards significance. For omics data, pathway enrichment analysis was performed. Pathways with *p* < 0.05 were considered significantly enriched, whereas those with 0.05 ≤ *p* < 0.10 were regarded as showing a tendency toward enrichment.

## 3. Results

### 3.1. Effects of NFF on Antioxidant Status in Serum, Liver, Spleen, and Thymus of Cashmere Goats

As presented in Table 2, at week 6 before morning feeding, the NFF group showed significantly higher serum levels of GPx, CAT, and T-AOC compared with the CON group (*p* = 0.012, *p* = 0.034, *p* = 0.017), along with lower MDA content (*p* = 0.011). No notable difference was found in T-SOD activity between the two groups (*p* = 0.440). At 3 h post-feeding, the NFF group exhibited significantly higher activities of GPx, T-SOD, CAT, and T-AOC relative to the CON group (*p* < 0.05), whereas MDA content was comparable between groups (*p* = 0.337). At week 12 before morning feeding (Table 3), GPx, CAT, and T-AOC activities in the serum of the NFF group were significantly higher than those in the CON group (*p* = 0.016, *p* = 0.012, *p* = 0.009), and the MDA content was significantly lower than that in the CON group (*p* = 0.005). At 3 h post-feeding, significantly lower activities of GPx, T-SOD, CAT, and T-AOC were observed in the NFF group than in the CON group (*p* < 0.05), whereas no significant intergroup difference was detected for MDA content (*p* = 0.458).

As detailed in Table 4, compared with the CON group, significant increases in GPx, T-AOC, and CAT activities were observed in the liver and thymus of the NFF group (*p* < 0.05), along with a borderline increase in liver T-SOD activity (*p* = 0.056) and a significant decrease in MDA content (*p* = 0.008). In the spleen, significantly higher activities of GPx, T-SOD, T-AOC, and CAT (*p* < 0.05) and lower MDA content (*p* = 0.035) were detected in the NFF group.

### 3.2. Effects of NFF on Inflammatory Parameters in Serum, Liver, Spleen, and Thymus of Cashmere Goats

According to Table 5, six weeks prior to the morning feeding, the serum levels of IL-2, IL-6, and LPS in the NFF group were notably lower compared to the CON group (*p* = 0.001, 0.009, and 0.014, respectively). Conversely, the IL-10 level was significantly elevated in the NFF group (*p* = 0.045). The levels of TNF-α and iNOS in the NFF group exhibited a downward trend. Three hours post-morning feeding, the levels of IL-1β, IL-2, IL-6, TNF-α, iNOS, and HIS were significantly reduced in the NFF group compared to the CON group (*p* < 0.05). The IL-10 level remained significantly higher (*p* = 0.021), and there was no significant difference in LPS levels between the groups (*p* = 0.439).

Table 6 presents serum inflammatory parameters at week 12 under two feeding conditions. Before morning feeding, the NFF group showed significantly lower levels of IL-1β, IL-2, IL-6, TNF-α, iNOS, HIS, and LPS (*p* < 0.05), along with higher IL-10 content (*p* = 0.029). At 3 h post-feeding, the pattern was similar for IL-1β, IL-2, IL-6, TNF-α, iNOS, and HIS (*p* < 0.05), and IL-10 remained elevated (*p* = 0.015).

As shown in Table 7, in comparison with the CON group, the NFF group exhibited a notable reduction in liver IL-1β, IL-2, and TNF-α levels (*p* = 0.001, *p* = 0.045, *p* = 0.006). There was a tendency for IL-6 and LPS levels in the liver to decrease (*p* = 0.058, *p* = 0.064). IL-10 levels in the liver, spleen, and thymus showed a significant increase (*p* = 0.035). The NFF group had significantly lower levels of thymus IL-1β, IL-2, IL-6, TNF-α, iNOS, and LPS compared with the CON group (*p* < 0.05).

### 3.3. Transcriptomic Analysis of the NFF Effects on the Liver of Cashmere Goat

#### 3.3.1. Sequencing Data Quality Control and Read Mapping

The quality control results of liver transcriptome sequencing data indicate that a total of 5.20 × 10^8^ raw reads were obtained during sequencing, while the purified reads (clean reads) yielded 5.14 × 10^8^, with each sample ranging from 4.05 × 10^7^ to 4.70 × 10^7^. The proportion of Q20 bases in the sequencing data was above 98%, while the proportion of Q30 bases was above 95%. The GC content was approximately 50%, and the average base error rate was below 0.0234%. All these results were within the normal range, indicating that the sequencing data were accurate and reliable, of high quality, and met the requirements for library construction, and could be used for subsequent analysis.

The comparison of clean reads from liver sequencing with the goat reference genome revealed that the total mapping ranged from 93.88% to 97.72%. Multiple mapping was observed between 5.97% and 8.78%, while unique mapping varied from 87.91% to 91.27%. These findings suggest that the libraries created for all liver samples were of high quality and suitable for further analysis.

#### 3.3.2. Differential Expression Genes (DEGs) and KEGG Pathway Enrichment Analysis

**Figure 1:**
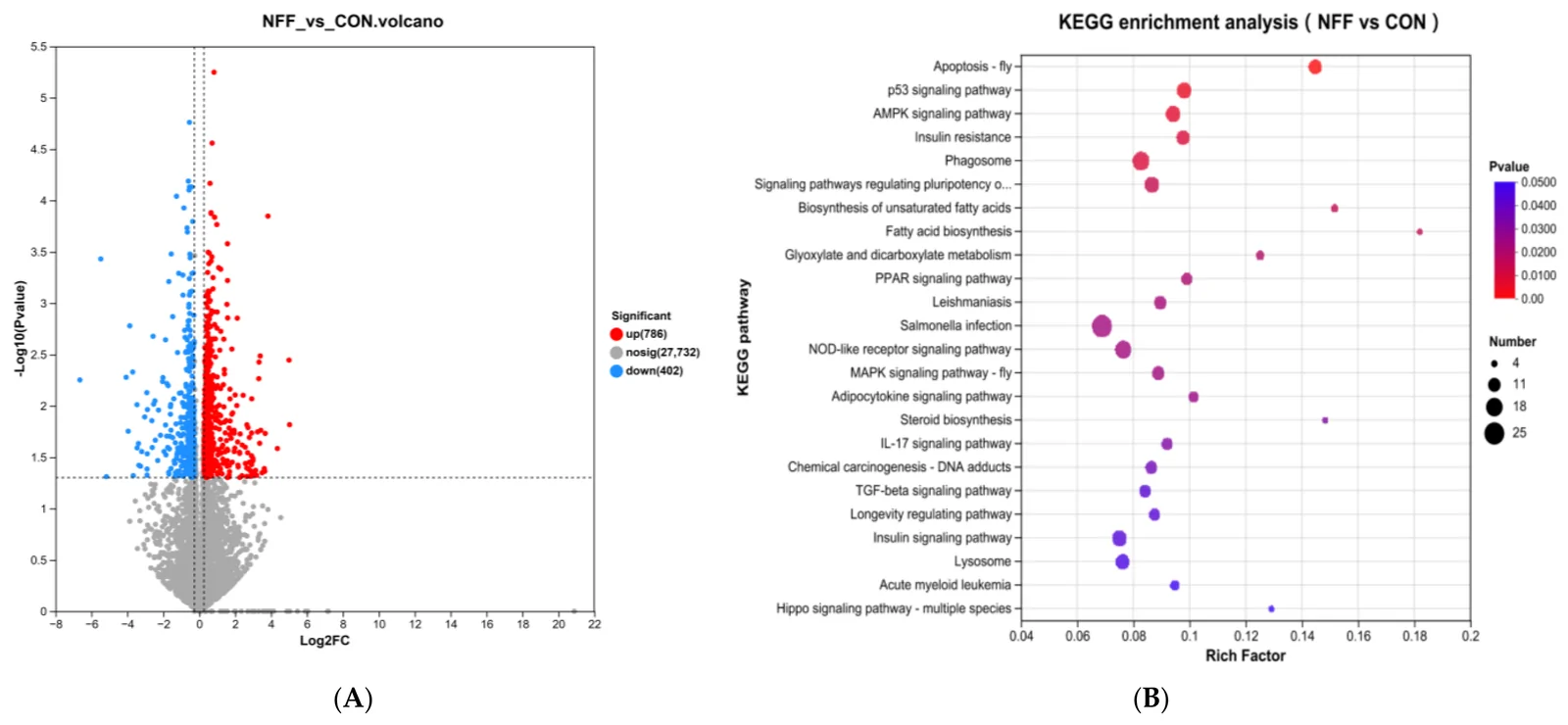
Volcano plot of DEGs and KEGG enrichment analysis. (**A**) Each point represents a specific gene. Red points indicate genes with significantly upregulated expression, blue points indicate genes with significantly downregulated expression, and gray points represent genes with no significant difference. Dotted lines indicate the thresholds for significant differential expression (|log_2_FC| ≥ log_2_(1.2), *p*-value < 0.05). (**B**) Shows the KEGG pathway enrichment analysis of DEGs between groups CON and NFF (top 30). CON = control (basal diet), NFF = noni fruit flavonoids (basal diet with 0.1% NFF).

KEGG pathway enrichment analysis was conducted on the DEGs derived from the comparative study of NFF and CON. This analysis revealed 327 pathways in total, with 24 of them showing significant enrichment. The top 30 pathways based on significance ranking are shown in Figure 1. The pathways related to antioxidant and anti-inflammatory responses that were enriched are presented in Table 8. Among them, there were 4 significantly enriched pathways (*p* < 0.05), including the p53 signaling pathway, NOD-like receptor signaling pathway, AMPK signaling pathway, and IL-17 signaling pathway; there were 3 pathways with enrichment tendencies (*p* = 0.050, *p* = 0.059, *p* = 0.094). In addition, 3 pathways showed a trend toward significant enrichment, namely Glutathione metabolism, TNF signaling pathway, and Thyroid hormone synthesis, respectively (*p* = 0.050, *p* = 0.059, and *p* = 0.094).

### 3.4. Validation of Transcriptome Sequencing Data

The relative expression levels of the 8 DEGs detected by qRT-PCR are shown in Table 9. The table, the variation patterns among various genomes are consistent with the transcriptome results, indicating that the transcriptome sequencing data at this time are reliable and effective.

### 3.5. Untargeted Liquid Chromatography–MS Metabolomic Analysis of the Effect of NFF on the Liver of Cashmere Goats Liver

#### 3.5.1. OPLS-DA Analysis

**Figure 2:**
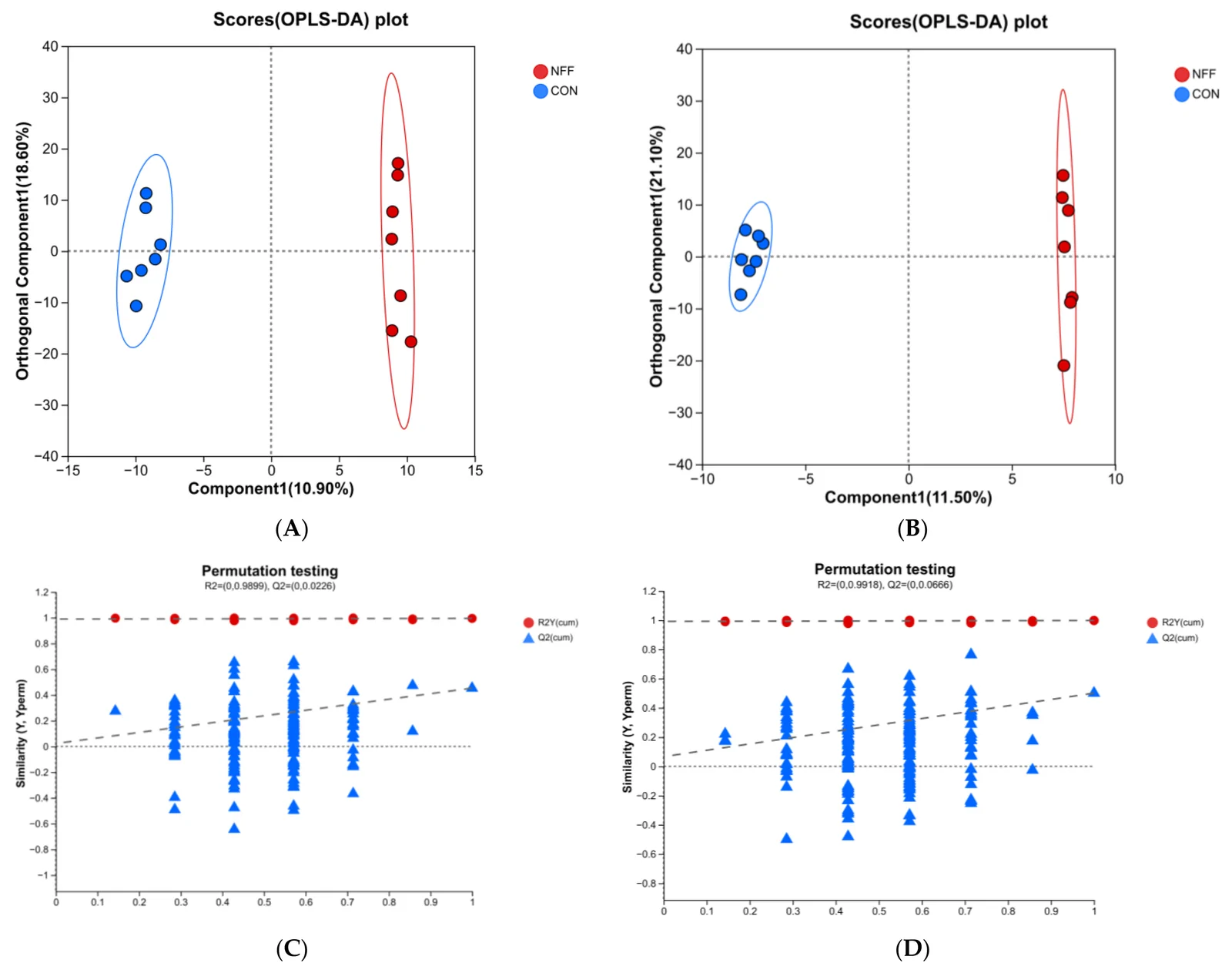
Analysis of liver non-targeted metabolite OPLS-DA. OPLS-DA under positive (ESI+) and negative (ESI−) electrospray ionization modes is shown in (**A**,**B**), and the corresponding substitution test results in (**C**,**D**). In (**C**,**D**), dashed lines indicate the linear regression fits for R^2^Y and Q^2^Y.

The analysis of the liver non-targeted metabolite OPLS-DA is shown in Figure 2. As can be seen from Figure 2A,B, the samples in the CON group and the NFF group have a high degree of aggregation, no crossover or overlap, and there is a significant difference in the composition of metabolites between the two groups. Figure 2C,D are mainly used to evaluate the rationality and reliability of OPLS-DA analysis. In this experiment, the R^2^Y values were 0.995 and 0.998, respectively, demonstrating excellent fitting performance to the original sample datasets. The original model Q^2^ values reached 0.501 and 0.453, which suggested satisfactory predictive capability of the constructed models. In addition, permutation verification was performed to exclude overfitting: the intercepts of permuted Q^2^ were 0.0226 and 0.0666 for cation and anion groups, respectively, with most randomly permuted Q^2^ points distributed below the zero line, further confirming the reliability of the established OPLS-DA models without obvious overfitting.

#### 3.5.2. Differential Metabolites and KEGG Pathway Enrichment Analysis

**Figure 3:**
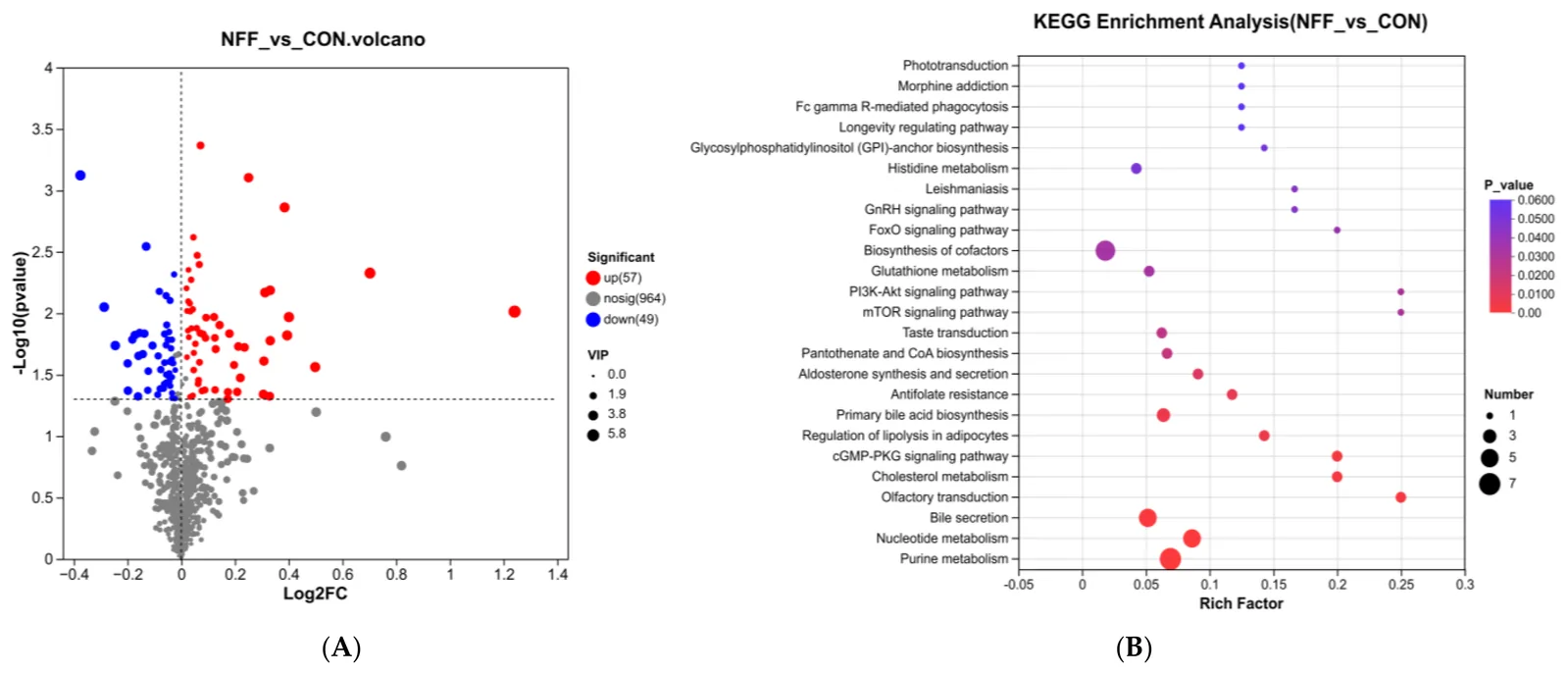
Volcano plot of differential metabolites and bubble plot for KEGG pathway enrichment analysis. (**A**) Each point represents a metabolite. The red points indicate metabolites with significantly increased expression levels, the blue points indicate metabolites with significantly decreased expression levels, and the gray points represent metabolites with no significant differences. Dotted lines indicate the thresholds for significant differential expression (|log_2_FC| ≥ log_2_(1), *p*-value < 0.05). (**B**) Present the KEGG pathway enrichment analysis results of the differential metabolites between the CON group and the NFF group (the top 25). CON = control (basal diet), NFF = noni fruit flavonoids (basal diet with 0.1% NFF).

The volcano plot of differential metabolites is shown in Figure 3A. Compared with the CON group, 57 differential metabolites were significantly upregulated, and 49 differential metabolites were significantly downregulated in the NFF group. KEGG pathway enrichment analysis was conducted on the differential metabolites, and a total of 63 pathways were obtained, among which 20 pathways were significantly enriched. The top 25 pathways ranked by significance are shown in Figure 3B. Enriched pathways related to antioxidation and anti-inflammation, along with the key metabolites within these pathways, are summarized in Table 10. Among them, purine metabolism, nucleotide metabolism, mTOR signaling pathways, glutathione metabolism, and biosynthesis of cofactors were significantly enriched (*p* < 0.05). Longevity-regulating pathways showed a significant enrichment trend (*p* = 0.058). Within these pathways, we specifically focused on three upregulated metabolites (Adenylosuccinate, Adenosine 5′-Monophosphate (AMP), N6-(1,2-Dicarboxyethyl)-AMP) and two downregulated metabolites (Cysteinylglycine, Pyroglutamic Acid), which are hypothesized to play pivotal roles in mediating the observed antioxidant and anti-inflammatory effects of NFF.

## 4. Discussion

Free radicals, by-products of oxidative metabolism, cause oxidative damage when antioxidant defenses are overwhelmed, thereby promoting chronic inflammation and diseases. Antioxidant enzymes (GPx, CAT, SOD) scavenge excess free radicals and maintain redox homeostasis. Lipid peroxidation, reflected by MDA levels, marks the extent of oxidative injury, while T-AOC gauges the systemic ability to counteract oxidative stress [15]. On the inflammatory front, unchecked free radicals activate nuclear factor kappa-light-chain-enhancer of activated B cells (NF-κB) signaling, driving transcription of pro-inflammatory mediators (TNF-α, iNOS, IL-6, IL-1β, IL-2) and triggering apoptotic programs. These factors fuel self-amplifying cascades and are also inducible by LPS and HIS. As an anti-inflammatory factor, IL-10 can inhibit inflammation and maintain homeostasis [16]. During the experimental period, dietary 0.1% NFF supplementation in cashmere goats enhanced T-AOC, GPx, CAT, T-SOD activities, elevated IL-10 content, and reduced the concentrations of MDA, IL-2, IL-6, iNOS, and TNF-α in serum. Serum IL-1β decreased significantly before morning feeding at week 6, and both before and 3 h after morning feeding at 12 weeks. The liver, spleen, and thymus are central organs for antioxidant and immune regulation, maintaining systemic homeostasis through metabolic detoxification, immune response mediation, and immune cell development and maturation, respectively. These results showed that NFF increased the activities of antioxidant enzymes and contents of anti-inflammatory factors in all three organs, while decreasing levels of lipid peroxides and pro-inflammatory factors. These changes were similar to those in serum. Therefore, dietary NFF supplementation improves antioxidant status, reduces lipid peroxidation, elevates anti-inflammatory mediators, and suppresses the pro-inflammatory factors and abnormal metabolites, thereby enhancing the anti-inflammatory capacity of cashmere goats. Although plant-derived flavonoids have been widely reported to improve antioxidant and anti-inflammatory function in animals, studies on NFF remain limited. For example, dietary rutin significantly elevated serum GPx, T-AOC, T-SOD, and CAT levels while reducing MDA levels in postpartum Hu sheep, thereby alleviating oxidative stress [17]. Moringa leaf flavonoids enhanced blood T-AOC and T-SOD activities and reduced MDA levels in dairy cows [18]. It has been reported that six flavonoids attenuate the D-galactose-induced decline of SOD, GPx, CAT, and T-AOC in mouse serum and liver, and enhance the expression of genes encoding antioxidant enzymes in the liver [19].

To understand how NFF simultaneously enhanced both antioxidant and anti-inflammatory markers, we performed integrated hepatic metabolomic and transcriptomic analyses. Metabolomic profiling identified significant increases in purine metabolism, nucleotide metabolism, and biosynthesis of cofactors, as evidenced by elevated AMP, adenylosuccinate, and N6-(1,2-dicarboxyethyl)-AMP. Additionally, AMP was specifically enriched in the mTOR and AMPK signaling pathways. Concurrently, transcriptome analysis revealed significant upregulation of *PRKAA1*, *PRKAA2*, and *PPARGC1A* within the AMPK signaling pathway. AMP functions as a crucial cellular energy sensor, maintaining homeostasis by modulating metabolic and signaling cascades. Adenylosuccinate and N^6^-(1,2-dicarboxyethyl)-AMP denote the same compound, which is cleaved by adenylosuccinate lyase to generate AMP and fumarate. [20,21]. An increase in AMP levels elevates the ratio of AMP/ATP ratio, which triggers the activation of AMP-activated protein kinase (AMPK) through the phosphorylation of Thr172 on its catalytic α subunit, encoded by *PRKAA1* and *PRKAA2* [22]. Once activated, AMPK stimulates nuclear factor erythroid 2-related factor 2 (Nrf2) to upregulate antioxidant enzymes (GPx, SOD, and CAT) and simultaneously inhibits NF-κB, reducing the release of pro-inflammatory factors (TNF-α, IL-1β, and IL-6). AMPK also directly interacts with peroxisome proliferator-activated receptor gamma coactivator 1α (PGC-1α), encoded by the *PPARGC1A*. In recent years, the AMPK/PGC-1α signaling pathway has been identified as a significant target for cancer therapy [23]. Studies suggest that resveratrol, a plant polyphenol similar to flavonoids, activates PGC-1α through *PRKAA1*, thereby influencing mitochondrial biogenesis in bovine mammary epithelial cells. This process suppresses reactive oxygen species (ROS) production, enhances cellular antioxidant capacity, and consequently alleviates inflammatory responses [24]. Previous studies have demonstrated that natural flavonoids constitute a significant class of AMPK activators. For example, quercetin, genistein, and aspalathin have been reported to activate AMPK. [25,26,27]. Rutin, as a dietary flavonoid, increases the activity of AMPK in hepatocytes [28]. Research on its anti-inflammatory properties indicates that quercetin enhances the expression of the AMPKα1/SIRT1 signaling pathway to mitigate macrophage-mediated inflammatory responses [29]. Quercetin has also been shown to activate the AMPK pathway, which then triggers downstream signaling and inhibits the mTOR and NF-κB pathways, thereby regulating autophagy activation, inflammatory response, and tumor metastasis [30,31]. Research on antioxidant effects revealed that kaempferol treatment increased AMPK phosphorylation levels in carbon tetrachloride-exposed rats, enhanced Nrf2 translocation to the nucleus, and upregulated hepatic heme oxygenase-1 expression, thereby improving oxidative balance in rat livers [32]. A study found that the flavonoid baicalin exhibits a highly positive correlation with N6-(1,2-dicarboxyethyl)-AMP, a potential biomarker associated with the antipyretic mechanism of a fever-reducing drug [33]. In the present study, qRT-PCR confirmed the upregulation of *PRKAA2* and *PPARGC1A*, consistent with the transcriptomic data. Collectively, the above results indicate that NFF may activate the AMPK signaling pathway by increasing the level of AMP, thereby regulating downstream antioxidant and anti-inflammatory responses.

Based on the above changes, we further analyzed the effects of NFF on antioxidant-related pathways and their downstream genes and metabolites. Hepatic metabolomics revealed significantly decreased cysteinylglycine and pyroglutamic acid levels within the glutathione (GSH) metabolism pathway in the NFF group. Transcriptomic analysis of the liver identified *GPX3*, *GPX8*, and *GCLM* as significantly upregulated genes in this pathway, and qRT-PCR confirmed these changes. Interpreting these metabolomic shifts requires consideration of GSH turnover dynamics. Cysteinylglycine is generated from GSH by γ-glutamyl transpeptidase during extracellular GSH breakdown, whereas pyroglutamic acid is produced from γ-glutamyl intermediates via the γ-glutamyl cycle when GSH synthesis is active [34]. Therefore, the concurrent decrease in both metabolites points to diminished flux through the degradation arm of the cycle. Importantly, this interpretation is mechanistically supported by the upregulation of *GCLM*, which encodes the modifier subunit of glutamate–cysteine ligase—the rate-limiting enzyme for GSH biosynthesis [35]. Increased *GCLM* expression favors enhanced synthetic capacity, providing sufficient reducing equivalents for GPx. Thus, the observed metabolic pattern suggests in the current study that NFF shifts glutathione metabolism toward enhanced synthesis and reduced turnover. This metabolic adaptation, together with the upregulation of *GPX3* and *GPX8*, culminates in elevated GPx activity and decreased lipid peroxidation. Moreover, some studies have shown that cysteinylglycine is an oxidant inducer that can induce oxidative stress and lipid peroxidation reactions under certain conditions [36], so the accumulation of cysteinylglycine may also cause an enhancement of oxidative stress response. Among the upregulated GPx isoforms, GPX3 encodes the primary free radical scavenger in plasma, and its enhanced expression suppresses liver cancer cell growth and metastasis [37], while GPX8 facilitates endoplasmic reticulum protein oxidative folding and participates in calcium homeostasis, thereby mitigating oxidative stress [38]. Taken together, NFF may enhance peroxide scavenging capacity by upregulating genes related to GPx and GSH synthesis while reducing GSH turnover, as indicated by decreased degradation intermediates and increased biosynthetic gene expression. These coordinated changes may culminate in markedly improved antioxidant defense.

Flavonoids exert anti-inflammatory effects by attenuating multiple signaling pathways associated with inflammation, cell proliferation, and apoptosis, with NF-κB inhibition serving as a central mechanism. In this study, transcriptomic analysis revealed that *NFKB1* (NF-κB1) and *IL1B* (IL-1β) in the NFF group were significantly downregulated in three highly enriched pathways: the IL-17 signaling pathway, NOD-like receptor signaling pathway, and the TNF signaling pathway. NF-κB1 (p50), part of the NF-κB family, plays a role in controlling inflammation, immune responses, cell growth, and programmed cell death, making it a possible target for therapies aimed at treating inflammation and cancer [39]. These findings suggest NFF may reduce hepatic IL-1B expression through modulating *NF-κB1.* The research also demonstrated a notable decrease in *CASP3* (caspase-3) expression within the IL-17, TNF, and p53 signaling pathways, as well as a marked reduction in *BID* (Bid) specifically in the p53 signaling pathway. CASP3 plays a crucial role in apoptosis and is intimately linked to inflammatory processes in living organisms [40]. During apoptotic signaling, caspase-8 activates Bid, a pro-apoptotic Bcl-2 family member, which then translocates to mitochondria, triggering cytochrome C release and subsequent caspase-3 activation [41]. The research indicates that programmed cell death plays a crucial role in completing inflammatory responses, and CASP inhibitors may serve as potential regulatory strategies for acute and chronic inflammatory diseases [42,43]. In conclusion, the downregulation of both *BID* and *CASP3* therefore suggests that NFF may attenuate inflammation-associated apoptosis. Additionally, *NLRP12* gene expression was significantly upregulated in the NOD-like receptor signaling pathway. As a regulatory protein with dual roles in inflammasome assembly and anti-inflammatory functions, NLRP12 reduces pro-inflammatory factor production by inhibiting NF-κB and ERK pathways [44]. NFF may indirectly inhibit *NFKB1* and *IL1B* by upregulating *NLRP12* and downregulating pro-apoptotic genes. Furthermore, within the TGF-β signaling pathway, *FST* (follistatin) was significantly upregulated while *ACVR1C* (activin A receptor) was significantly downregulated. Activin A signals through the ACVR1C receptor, and its expression correlates closely with inflammation severity [45]. The endogenous antagonist follistatin blocks this pathway by competitively binding activin A, thereby mitigating inflammatory injury [46]. These results indicate that NFF simultaneously promotes FST-mediated activin A sequestration and suppresses ACVR1C expression, providing two complementary mechanisms for TGF-β pathway blockade. qRT-PCR validation confirmed the transcriptomic changes in *NFKB1*, *IL1B*, *CASP3*, *BID*, *NLRP12*, and *ACVR1C*. Taken together, NFF exerts its anti-inflammatory effects probably through a multi-pathway network encompassing suppression of NF-κB and TGF-β signaling, upregulation of NLRP12, and attenuation of apoptotic pathways.

**Figure 4:**
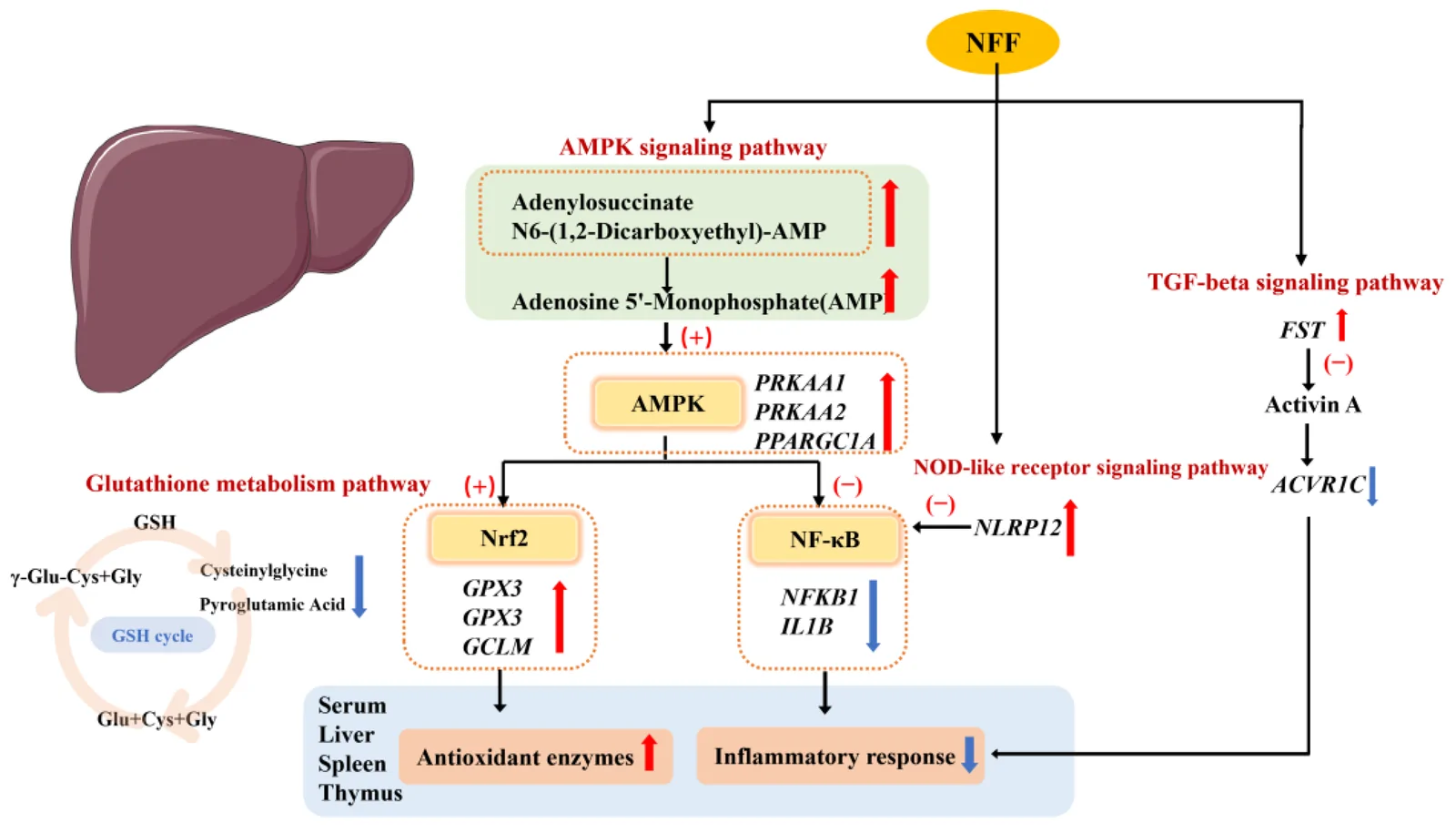
Mechanism diagram. Abbreviations: NFF = Noni fruit flavonoids; AMPK = adenosine monophosphate-activated protein kinase; Nrf2: nuclear factor erythroid 2-related factor 2; NF-κB: nuclear factor kappa-light-chain-enhancer of activated B cells; *PRKAA* = protein kinase AMP-activated catalytic subunit alpha; *PPARGC1A* = peroxisome proliferator-activated receptor gamma coactivator 1-alpha; *GCLM* = glutamate–cysteine ligase, modulatory subunit; *GPX* = glutathione peroxidase; *NFKB1* = nuclear factor kappa B subunit 1; *NLRP12* = NOD-like receptor family pyrin domain containing 12; *IL1B* = interleukin-1 beta; *FST*: follistatin; *ACVR1C*: activin A receptor type 1C; GSH: glutathione; Glu: glutamic acid; Cys: cysteine; Gly: glycine. “(+)” indicates activation, and “(−)” indicates inhibition. “⬆” indicates a significant increase, and “⬇” indicates a significant decrease. The liver schematic was sourced from Servier Medical Art (SMART) (https://smart.servier.com/), available under the CC BY 4.0 license.

Collectively, this study reveals the correlation between AMPK activation and the differential expression of genes such as *GPX3*, *GPX8*, *GCLM*, *NFKB1*, and *IL1B*. These genes are regulated by the Nrf2 and NF-κB signaling pathways, respectively. *GPX3*, *GPX8*, and *GCLM* are typical downstream antioxidant target genes of the Nrf2 pathway, while *NFKB1* serves as a core component of the NF-κB pathway involved in the regulation of inflammation. This finding suggests that AMPK may synergistically regulate the expression of genes related to antioxidation and anti-inflammation by simultaneously modulating both the Nrf2 and NF-κB pathways. The possible mechanism is shown in Figure 4. However, several limitations of this study should be acknowledged. The sample size was relatively small, although this is not uncommon in large-animal studies given practical constraints. Additionally, protein-level validation was not performed, targeted metabolite confirmation was not conducted, and functional pathway experiments were not carried out to establish causal relationships. Further studies with larger sample sizes, protein validation, targeted metabolite verification, and functional assays are needed to validate and extend our findings.

## 5. Conclusions

Dietary supplementation with 0.1% NFF effectively enhanced the antioxidant status and anti-inflammatory response in cashmere goats. Integrative transcriptomic and metabolomic analyses suggest that the AMPK signaling pathway, glutathione metabolism, and TGF-β signaling pathway may be critically involved in mediating these effects. *GPX3*, *GPX8*, *GCLM*, *NFKB1*, and *IL1B* were identified as key candidate genes, while AMP, cysteinylglycine, and pyroglutamic acid were key differential metabolites. These findings provide a foundation for future mechanistic studies, though the sample size, lack of protein validation, and absence of functional experiments warrant caution.

## Figures and Tables

**Table 1:** Ingredients and nutrient levels of the diets (air-dry basis).

Item	Weeks 1–6	Weeks 7–12
Ingredients (%)		
Alfalfa	20.00	32.00
Corn stalk	24.00	16.00
Oat hay	36.00	32.00
Corn	1.00	10.80
Soybean meal	13.50	4.25
Bran	0.90	0.70
Corn germmeal	1.10	0.50
Soybean oil	0.70	1.50
Premix ^1^	0.50	0.50
Limestone	1.00	0.20
CaHPO4	0.90	1.15
NaCl	0.40	0.40
Total	100.00	100.00
Nutrient levels ^2^		
DE (MJ/Kg)	11.16	11.83
DM (%)	93.68	94.67
CP (%)	14.00	12.01
EE (%)	2.46	3.57
Non-structural carbohydrates (%)	23.68	31.40
NDF (%)	46.48	43.16
ADF (%)	26.15	24.52
Ca (%)	0.98	0.84
P (%)	0.40	0.42

^1^ The premix provided the following per kg of diet: Fe 40 mg, Cu 8 mg, Zn 50 mg, Mn 30 mg, I 0.3 mg, Se 0.3 mg, Co 0.25 mg, VA 6000 IU, VD3 2500 IU, VE 12.5 IU, VK3 1.8 mg VB1 0.035 mg, VB2 8.5 mg, VB6 0.9 mg, nicotinic acid 22 mg, D-pantothenic acid 17 mg, VB12 0.03 mg, biotin 0.14 mg folic acid 1.5 mg. ^2^ The digestible energy value was calculated, while the remaining nutrient values were the measured values.

**Table 2:** Effects of NFF on serum antioxidant status in cashmere goats at week 6.

Items	CON ^1^	NFF ^1^	SEM ^2^	*p*-Value
Before morning feeding				
T-AOC (U/mL)	1.97 ^b^	2.48 ^a^	0.163	0.017
T-SOD (U/mL)	103.21 ^a^	101.57 ^a^	2.005	0.440
GPx (U/mL)	278.50 ^b^	295.03 ^a^	4.888	0.012
CAT (U/mL)	3.68 ^b^	4.25 ^a^	0.218	0.034
MDA (nmol/mL)	1.43 ^a^	1.11 ^b^	0.081	0.011
After morning feeding 3 h				
T-AOC (U/mL)	1.28 ^b^	1.94 ^a^	0.140	0.001
T-SOD (U/mL)	100.96 ^b^	117.03 ^a^	3.353	0.011
GPx (U/mL)	314.39 ^b^	360.81 ^a^	12.505	0.008
CAT (U/mL)	0.52 ^b^	1.00 ^a^	0.125	0.007
MDA (nmol/mL)	1.22 ^a^	1.13 ^a^	0.054	0.337

^1^ CON = control (basal diet); NFF = noni fruit flavonoids (basal diet with 0.1% NFF). ^2^ SEM = standard error of the mean. ^a,b^ Different superscript letters within the same row indicate significant differences between experimental groups (*p* < 0.05). Abbreviations: T-AOC = total antioxidant capacity; T-SOD = total superoxide dismutase; GPx = glutathione peroxidase; CAT = catalase; MDA = malondialdehyde.

**Table 3:** Effects of NFF on serum antioxidant status in cashmere goats at week 12.

Items	CON ^1^	NFF ^1^	SEM ^2^	*p*-Value
Before morning feeding				
T-AOC (U/mL)	1.52 ^b^	1.93 ^a^	0.113	0.009
T-SOD (U/mL)	84.01 ^a^	84.37 ^a^	0.485	0.480
GPx (U/mL)	130.21 ^b^	193.96 ^a^	20.137	0.016
CAT (U/mL)	2.76 ^b^	3.17 ^a^	0.121	0.012
MDA (nmol/mL)	1.68 ^a^	1.30 ^b^	0.094	0.005
After morning feeding 3 h				
T-AOC (U/mL)	1.68 ^b^	2.48 ^a^	0.134	0.006
T-SOD (U/mL)	137.81 ^b^	150.59 ^a^	2.505	0.001
GPx (U/mL)	193.22 ^b^	242.41 ^a^	14.631	0.012
CAT (U/mL)	0.48 ^b^	0.71 ^a^	0.057	0.006
MDA (nmol/mL)	1.35 ^a^	1.40 ^a^	0.065	0.458

^1^ CON = control (basal diet); NFF = noni fruit flavonoids (basal diet with 0.1% NFF). ^2^ SEM = standard error of the mean. ^a,b^ Different superscript letters within the same row indicate significant differences between experimental groups (*p* < 0.05). Abbreviations: T-AOC = total antioxidant capacity; T-SOD = total superoxide dismutase; GPx = glutathione peroxidase; CAT = catalase; MDA = malondialdehyde.

**Table 4:** Effects of NFF on antioxidant status in liver, spleen, and thymus of cashmere goats.

Items	CON ^1^	NFF ^1^	SEM ^2^	*p*-Value
Liver				
GPx (U/mgprot)	56.90 ^b^	63.63 ^a^	2.629	0.037
T-SOD (U/mgprot)	719.90	831.55	48.668	0.056
T-AOC (U/mgprot)	1.13 ^b^	1.36 ^a^	0.048	0.002
CAT (U/mgprot)	97.17 ^b^	104.13 ^a^	1.708	0.003
MDA (nmol/mgprot)	2.48 ^a^	1.76 ^b^	0.177	0.008
Spleen				
GPx (U/mgprot)	161.04 ^b^	193.50 ^a^	12.487	0.036
T-SOD (U/mgprot)	99.52 ^b^	114.75 ^a^	3.655	0.004
T-AOC (U/mgprot)	0.58 ^b^	0.74 ^a^	0.027	0.006
CAT (U/mgprot)	3.85 ^b^	5.16 ^a^	0.367	0.009
MDA (nmol/mgprot)	1.69 ^a^	1.34 ^b^	0.102	0.035
Thymus				
GPx (U/mgprot)	597.75 ^b^	703.75 ^a^	43.117	0.044
T-SOD (U/mgprot)	33.38 ^a^	31.35 ^a^	3.058	0.529
T-AOC (U/mgprot)	2.21 ^b^	2.88 ^a^	0.225	0.021
CAT (U/mgprot)	2.80 ^b^	3.83 ^a^	0.435	0.050
MDA (nmol/mgprot)	1.50 ^a^	1.84 ^a^	0.404	0.423

^1^ CON = control (basal diet); NFF = noni fruit flavonoids (basal diet with 0.1% NFF). ^2^ SEM = standard error of the mean. ^a,b^ Different superscript letters within the same row indicate significant differences between experimental groups (*p* < 0.05). Abbreviations: T-AOC = total antioxidant capacity; T-SOD = total superoxide dismutase; GPx = glutathione peroxidase; CAT = catalase; MDA = malondialdehyde.

**Table 5:** Effects of NFF on serum inflammatory parameters in cashmere goats at week 6 (ng/mL).

Items	CON ^1^	NFF ^1^	SEM ^2^	*p*-Value
Before morning feeding				
IL-1β	14.58 ^a^	14.47 ^a^	0.862	0.903
IL-2	145.94 ^a^	100.41 ^b^	7.418	0.001
IL-6	15.78 ^a^	13.81 ^b^	0.544	0.009
IL-10	3.34 ^b^	4.12 ^a^	0.272	0.045
TNF-α	3.82	3.15	0.351	0.096
iNOS	5.96	5.35	0.302	0.082
HIS	3.75 ^a^	3.71 ^a^	0.112	0.671
LPS	106.60 ^a^	92.71 ^b^	4.300	0.014
After morning feeding 3 h				
IL-1β	14.99 ^a^	13.65 ^b^	0.427	0.022
IL-2	106.62 ^a^	91.40 ^b^	3.119	0.002
IL-6	16.98 ^a^	12.81 ^b^	1.157	0.015
IL-10	5.10 ^b^	5.60 ^a^	0.170	0.021
TNF-α	2.14 ^a^	1.81 ^b^	0.112	0.023
iNOS	3.73 ^a^	3.33 ^b^	0.092	0.003
HIS	3.24 ^a^	2.71 ^b^	0.191	0.028
LPS	92.95 ^a^	90.21 ^a^	3.342	0.439

^1^ CON = control (basal diet); NFF = noni fruit flavonoids (basal diet with 0.1% NFF). ^2^ SEM = standard error of the mean. ^a,b^ Different superscript letters within the same row indicate significant differences between experimental groups (*p* < 0.05). Abbreviations: IL = interleukin; TNF-α = tumor necrosis factor-alpha; iNOS = inducible nitric oxide synthase; HIS = histamine; LPS = lipopolysaccharide.

**Table 6:** Effects of NFF on serum inflammatory parameters in cashmere goats at week 12 (ng/mL).

Items	CON ^1^	NFF ^1^	SEM ^2^	*p*-Value
Before morning feeding				
IL-1β	17.77 ^a^	13.80 ^b^	1.155	0.011
IL-2	185.98 ^a^	144.27 ^b^	7.883	0.001
IL-6	25.15 ^a^	18.87 ^b^	1.132	0.001
IL-10	7.88 ^b^	8.65 ^a^	0.280	0.029
TNF-α	3.47 ^a^	3.02 ^b^	0.183	0.043
iNOS	4.91 ^a^	4.32 ^b^	0.167	0.010
HIS	5.00 ^a^	4.55 ^b^	0.154	0.024
LPS	115.65 ^a^	100.06 ^b^	4.451	0.010
After morning feeding 3 h				
IL-1β	9.99 ^a^	7.58 ^b^	0.654	0.008
IL-2	138.88 ^a^	120.75 ^b^	6.228	0.023
IL-6	24.12 ^a^	20.25 ^b^	1.201	0.015
IL-10	4.13 ^b^	4.97 ^a^	0.282	0.015
TNF-α	2.34	2.06	0.074	0.058
iNOS	4.29 ^a^	3.95 ^b^	0.082	0.004
HIS	4.99 ^a^	4.18 ^b^	0.172	0.002
LPS	137.07 ^a^	137.27 ^a^	7.621	0.980

^1^ CON = control (basal diet); NFF = noni fruit flavonoids (basal diet with 0.1% NFF). ^2^ SEM = standard error of the mean. ^a,b^ Different superscript letters within the same row indicate significant differences between experimental groups (*p* < 0.05). Abbreviations: IL = interleukin; TNF-α = tumor necrosis factor-alpha; iNOS = inducible nitric oxide synthase; HIS = histamine; LPS = lipopolysaccharide.

**Table 7:** Effects of NFF on inflammatory parameters in liver, spleen, and thymus of cashmere goats (ng/mgprot).

Items	CON ^1^	NFF ^1^	SEM ^2^	*p*-Value
Liver				
IL-1β	0.53 ^a^	0.43 ^b^	0.018	0.001
IL-2	2.13 ^a^	1.88 ^b^	0.099	0.045
IL-6	0.40	0.33	0.021	0.058
IL-10	0.11 ^b^	0.13 ^a^	0.006	0.035
TNF-α	0.73 ^a^	0.66 ^b^	0.018	0.006
iNOS	0.04 ^a^	0.04 ^a^	0.005	0.544
HIS	0.08 ^a^	0.07 ^a^	0.005	0.141
LPS	1.89	1.75	0.060	0.064
Spleen				
IL-1β	0.85 ^a^	0.84 ^a^	0.035	0.788
IL-2	4.20 ^a^	4.09 ^a^	0.240	0.679
IL-6	0.61 ^a^	0.60 ^a^	0.018	0.751
IL-10	0.21 ^b^	0.25 ^a^	0.009	0.005
TNF-α	1.36 ^a^	1.26 ^b^	0.037	0.024
iNOS	0.08 ^a^	0.08 ^a^	0.007	0.564
HIS	0.14 ^a^	0.13 ^a^	0.006	0.153
LPS	2.99 ^a^	2.90 ^a^	0.178	0.620
Thymus				
IL-1β	1.93 ^a^	1.54 ^b^	0.053	<0.001
IL-2	8.79 ^a^	7.26 ^b^	0.318	0.002
IL-6	1.29 ^a^	0.95 ^b^	0.141	0.045
IL-10	0.38 ^b^	0.49 ^a^	0.038	0.026
TNF-α	2.50 ^a^	1.98 ^b^	0.067	<0.001
iNOS	0.16 ^a^	0.10 ^b^	0.010	0.001
HIS	0.28 ^a^	0.24 ^a^	0.031	0.288
LPS	6.37 ^a^	4.84 ^b^	0.123	<0.001

^1^ CON = control (basal diet); NFF = noni fruit flavonoids (basal diet with 0.1% NFF). ^2^ SEM = standard error of the mean. ^a,b^ Different superscript letters within the same row indicate significant differences between experimental groups (*p* < 0.05). Abbreviations: IL = interleukin; TNF-α = tumor necrosis factor-alpha; iNOS = inducible nitric oxide synthase; HIS = histamine; LPS = lipopolysaccharide.

**Table 8:** Partial KEGG pathways enriched by liver transcriptome (NFF vs. CON).

Pathway id	Pathway Name	*p*-Value	Partially Up-Regulated Genes ^1^	Partially Down-Regulated Genes ^2^
map04115	p53 signaling pathway	0.005		*BID*, *CASP3*
map04152	AMPK signaling pathway	0.007	*PRKAA1*, *PRKAA2*, *PPARGC1A*	
map04621	NOD-like receptor signaling pathway	0.023	*NLRP12*	*IL1B*, *NFKB1*
map04657	IL-17 signaling pathway	0.031		*NFKB1*, *IL1B*, *CASP3*
map00480	Glutathione metabolism	0.050	*GPX8*, *GPX3*, *GCLM*	
map04350	TGF-beta signaling pathway	0.041	*FST*	*ACVR1C*
map04668	TNF signaling pathway	0.059		*NFKB1*, *IL1B*, *CASP3*
map04918	Thyroid hormone synthesis	0.094	*GPX8*, *GPX3*	
map04210	Apoptosis	0.334		*NFKB1*, *BID*, *CASP3*
map04064	NF-kappa B signaling pathway	0.377		*NFKB1*, *IL1B*

^1^ Partial list of significantly upregulated genes in the liver of NFF vs. CON cashmere goats. ^2^ Partial list of significantly downregulated genes in the liver of NFF vs. CON cashmere goats. CON = control (basal diet), NFF = noni fruit flavonoids (basal diet with 0.1% NFF). Abbreviations: *PRKAA* = protein kinase AMP-activated catalytic subunit alpha; *PPARGC1A* = peroxisome proliferator-activated receptor gamma coactivator 1-alpha; *GCLM* = glutamate–cysteine ligase, modulatory subunit; *GPX* = glutathione peroxidase; *NFKB1* = nuclear factor kappa B subunit 1; *NLRP12* = NOD-like receptor family pyrin domain containing 12; *IL1B* = interleukin-1 beta; *CASP3* = caspase-3; *FST* = follistatin; *ACVR1C* = activin A receptor type 1C.

**Table 9:** Effect of NFF on the expression of liver-related genes in cashmere goats.

Item	CON ^1^	NFF ^1^	SEM ^2^	*p*-Value
*GCLM*	1.00 ^b^	1.92 ^a^	0.174	0.004
*GPX8*	1.00 ^b^	1.71 ^a^	0.128	0.003
*GPX3*	1.00 ^b^	2.01 ^a^	0.096	<0.001
*PPARGC1A*	1.00 ^b^	3.27 ^a^	0.696	0.002
*PRKAA2*	1.00 ^b^	1.72 ^a^	0.140	0.002
*NFKB1*	1.00 ^a^	0.47 ^b^	0.059	<0.001
*NLRP12*	1.00 ^b^	2.58 ^a^	0.580	0.001
*IL-1B*	1.00 ^a^	0.62 ^b^	0.044	<0.001
*CASP3*	1.00 ^a^	0.72 ^b^	0.040	<0.001
*ACVR1C*	1.00 ^a^	0.38 ^b^	0.080	0.001

^1^ CON = control (basal diet); NFF = noni fruit flavonoids (basal diet with 0.1% NFF). ^2^ SEM = standard error of the mean. ^a,b^ Different superscript letters within the same row indicate significant differences between experimental groups (*p* < 0.05). Abbreviations: *GCLM* = glutamate–cysteine ligase, modulatory subunit; *GPX* = glutathione peroxidase; *PPARGC1A* = peroxisome proliferator-activated receptor gamma coactivator 1-alpha; *PRKAA2* = protein kinase AMP-activated catalytic subunit alpha 2; *NFKB1* = nuclear factor kappa B subunit 1; *NLRP12* = NOD-like receptor family pyrin domain containing 12; *IL1B* = interleukin-1 beta; *CASP3* = caspase-3; *ACVR1C* = activin A receptor type 1C.

**Table 10:** Partial KEGG pathways enriched with differential metabolites in the liver (NFF vs. CON).

Pathway id	Pathway Name	*p*-Value	Partially Up-Regulated Genes ^1^	Partially Down-Regulated Genes ^2^
map00230	Purine metabolism	<0.01	Adenylosuccinate, Adenosine 5′-Monophosphate, N6-(1,2-Dicarboxyethyl)-AMP	
map01232	Nucleotide metabolism	<0.01	Adenylosuccinate, Adenosine 5′-Monophosphate, N6-(1,2-Dicarboxyethyl)-AMP	
map04150	mTOR signaling pathway	0.030	Adenosine 5′-Monophosphate	
map00480	Glutathione metabolism	0.032		Cysteinylglycine, Pyroglutamic Acid
map01240	Biosynthesis of cofactors	0.032	Adenylosuccinate, Adenosine 5′-Monophosphate, N6-(1,2-Dicarboxyethyl)-AMP	
map04211	Longevity regulating pathway	0.058	Adenosine 5′-Monophosphate	
map04152	AMPK signaling pathway	0.153	Adenosine 5′-Monophosphate	

^1^ Partial list of significantly upregulated metabolites in the liver of NFF vs. CON cashmere goats. ^2^ Partial list of significantly downregulated metabolites in the liver of NFF vs. CON cashmere goats. CON = control (basal diet), NFF = noni fruit flavonoids (basal diet with 0.1% NFF).

## Data Availability

The original data presented in the study are openly available in the China National GeneBank Database (CNGBdb) at accession number CNP0009688.

## Supplementary Materials

The following supporting information can be downloaded at https://www.mdpi.com/article/10.3390/antiox15080987/s1, Table S1: Candidate gene primer sequences.

## Abbreviations

The following abbreviations are used in this manuscript:

NFF	Noni fruit flavonoids
CON	Control
T-AOC	Total antioxidant capacity
GPx	Glutathione peroxidase
CAT	Catalase
IL	Interleukin
TNF-α	Tumor necrosis factor-α
AMP	Adenosine-5′-monophosphate
LPS	Lipopolysaccharide
HIS	Histamine
SOD	Superoxide dismutase
iNOS	Inducible nitric oxide synthase
*PRKAA*	Protein kinase AMP-activated catalytic subunit alpha
*PPARGC1A*	Peroxisome proliferator-activated receptor gamma coactivator 1-alpha
*GCLM*	Glutamate–cysteine ligase, modulatory subunit
*NFKB1*	Nuclear factor kappa B subunit 1
*NLRP12*	NOD-like receptor family pyrin domain containing 12
*IL1B*	Interleukin-1 beta
*FST*	Follistatin
*ACVR1C*	Activin A receptor type 1cC
*CASP*	Caspase
ROS	Reactive oxygen species

## References

[1] Wang X.Y., Shen W., Wu P., Wang C.L., Li J.H., Wang D., Yue W.F. (2024). How food consumption trends change the direction of sheep breeding in China. Animals.

[2] Kemp D.R., Behrendt K., Badgery W.B., Han G.D., Li P., Zhang Y., Wu J., Hou F.J. (2020). Chinese degraded grasslands—Pathways for sustainability. Rangel. J..

[3] So-In C., Sunthamala N. (2023). Influence of goat management systems on hematological, oxidative stress profiles, and parasitic gastrointestinal infection. Vet. World.

[4] Zheng Q.H., Tan W.J., Feng X.L., Feng K.X., Zhong W.T., Liao C.Y., Liu Y.T., Li S.J., Hu W.Z. (2022). Protective effect of flavonoids from mulberry leaf on AAPH-induced oxidative damage in sheep erythrocytes. Molecules.

[5] Sun H., Zhao F.F., Hou F.Y., Jin Y.Q., Zhang X.Z., Ma Y., Zhang Y., Fan Y.T., Yang Z.Q., Wang H.R. (2022). Influences of naringin supplementation on ruminal fermentation, inflammatory response, antioxidant capacity and bacterial community in high-concentrate diet of fattening goats. Ital. J. Anim. Sci..

[6] Almeida É.S., de Oliveira D., Hotza D. (2019). Properties and applications of *Morinda citrifolia* (noni): A review. Compr. Rev. Food Sci. Food Saf..

[7] Hou S.L., Ma D.Y., Wu S.F., Hui Q.Y., Hao Z.H. (2025). *Morinda citrifolia* L.: A comprehensive review on phytochemistry, pharmacological effects, and antioxidant potential. Antioxidants.

[8] Zhang Q.Y., Dong S.H., Li Y.H., Zhao Y.L., Shi B.L., Yan S.M. (2022). Comparative study on antioxidant ability of extracts from noni (*Morinda citrifolia* L.) fruit with different solvents. J. Chin. Cereals Oils Assoc..

[9] Zhang Q.Y., Dong S.H., Li Y.H., Liu J.T., Zhang S.X., Yu H., Zhao Y.L., Guo X.Y., Guo Y.M., Shi B.L. (2024). Effects of noni fruit flavonoids on in vitro ruminal fermentation of cashmere goats with high-concentrate substrate. Chin. J. Anim. Nutr..

[10] Zhang Q.Y. (2023). Alleviating Effect and Mechanism of Noni Fruit Flavonoid on the Decline Ofantioxidant and Immune Function in Cashmere Goatsinduced by High Concentrate Diet. Ph.D. Thesis.

[11] Zhang Q.Y., Dong S.H., Yu H., Li Y.H., Guo X.Y., Zhao Y.L., Guo Y.M., Yan S.M. (2023). Effects of Noni (*Morinda citrifolia* L.) fruit extract supplemented in cashmere goats with a high-concentrate diet on growth performance, ruminal and colonic fermentation and sara. Animals.

[12] (2021). Nutrient Requirements of Meat-Type Sheep and Goat.

[13] Wang X., Martin G.B., Wen Q., Liu S.L., Zhang J., Yu Y., Shi B.L., Guo X.Y., Zhao Y.L., Yan S.M. (2019). Linseed oil and heated linseed grain supplements have different effects on rumen bacterial community structures and fatty acid profiles in cashmere kids1. J. Anim. Sci..

[14] Livak K.J., Schmittgen T.D. (2001). Analysis of relative gene expression data using real-time quantitative pcr and the 2^−ΔΔCT^ method. Methods.

[15] Jomova K., Raptova R., Alomar S.Y., Alwasel S.H., Nepovimova E., Kuca K., Valko M. (2023). Reactive oxygen species, toxicity, oxidative stress, and antioxidants: Chronic Diseases and Aging. Arch. Toxicol..

[16] Ma Q., Hao S., Hong W., Tergaonkar V., Sethi G., Tian Y., Duan C. (2024). Versatile function of NF-ĸB in inflammation and cancer. Exp. Hematol. Oncol..

[17] Ding H.Y., Li Y., Zhao C., Yang Y., Xiong C.K., Zhang D.L., Feng S.B., Wu J.J., Wang X.C. (2022). Rutin supplementation reduces oxidative stress, inflammation and apoptosis of mammary gland in sheep during the transition period. Front. Vet. Sci..

[18] Liu J., Wang Y., Liu L., Ma G.M., Zhang Y.G., Ren J. (2023). Effect of Moringa leaf flavonoids on the production performance, immune system, and rumen fermentation of dairy cows. Vet. Med. Sci..

[19] Zeng Y.X., Song J.J., Zhang M.M., Wang H.W., Zhang Y., Suo H.Y. (2020). Comparison of in vitro and in vivo antioxidant activities of six flavonoids with similar structures. Antioxidants.

[20] Dewulf J.P., Marie S., Nassogne M.-C. (2022). Disorders of purine biosynthesis metabolism. Mol. Genet. Metab..

[21] Steinberg G.R., Hardie D.G. (2023). New insights into activation and function of the AMPK. Nat. Rev. Mol. Cell Biol..

[22] Yang C., Rubin L., Yu X., Lazarovici P., Zheng W. (2024). Preclinical evidence using synthetic compounds and natural products indicates that AMPK represents a potential pharmacological target for the therapy of pulmonary diseases. Med. Res. Rev..

[23] Keerthana C.K., Rayginia T.P., Shifana S.C., Anto N.P., Kalimuthu K., Isakov N., Anto R.J. (2023). The role of AMPK in cancer metabolism and its impact on the immunomodulation of the tumor microenvironment. Front. Immunol..

[24] Hu C.L., An Y.H., Ma X.H., Feng X., Ma Y., Ma Y.F. (2025). Resveratrol activates PGC-1α pathway via PRAKK1 to regulate mitochondrial biogenesis and alleviate inflammatory responses in bovine mammary epithelial cells. Anim. Nutr..

[25] Krishnamoorthy R.M., Venkatraman A.C. (2017). Polyphenols activate energy sensing network in insulin resistant models. Chem. Biol. Interact..

[26] Ikeda T., Watanabe S., Mitani T. (2022). Genistein Regulates Adipogenesis by blocking the function of adenine nucleotide translocase-2 in the mitochondria. Biosci. Biotechnol. Biochem..

[27] Mazibuko-Mbeje S.E., Dludla P.V., Roux C., Johnson R., Ghoor S., Joubert E., Louw J., Opoku A.R., Muller C.J.F. (2019). Aspalathin-enriched green rooibos extract reduces hepatic insulin resistance by modulating PI3K/AKT and AMPK Pathways. Int. J. Mol. Sci..

[28] Wu C.H., Lin M.C., Wang H.C., Yang M.Y., Jou M.J., Wang C.J. (2011). Rutin inhibits oleic acid induced lipid accumulation via reducing lipogenesis and oxidative stress in hepatocarcinoma cells. J. Food Sci..

[29] Dong J., Zhang X., Zhang L., Bian H.X., Xu N., Bao B., Liu J. (2014). Quercetin reduces obesity-associated ATM infiltration and inflammation in mice: A mechanism including AMPKα1/SIRT1. J. Lipid Res..

[30] Li H., Chen C. (2018). Quercetin has antimetastatic effects on gastric cancer cells via the interruption of uPA/uPAR function by modulating NF-κb, PKC-δ, ERK1/2, and AMPKα. Integr. Cancer Ther..

[31] Rivera Rivera A., Castillo-Pichardo L., Gerena Y., Dharmawardhane S. (2016). Anti-breast cancer potential of quercetin via the Akt/AMPK/mammalian target of rapamycin (mTOR) signaling cascade. PLoS ONE.

[32] Lee C., Yoon S., Moon J.-O. (2023). Kaempferol suppresses carbon tetrachloride-induced liver damage in rats via the MAPKs/NF-κB and AMPK/Nrf2 signaling pathways. Int. J. Mol. Sci..

[33] Zhang Z.X., Lu F., Liu H.Y., Zhao H.Z., Liu Y.H., Fu S., Wang M.L., Xie Z.Y., Yu H.H., Huang Z.H. (2017). An integrated strategy by using target tissue metabolomics biomarkers as pharmacodynamic surrogate indices to screen antipyretic components of Qingkaikling injection. Sci. Rep..

[34] Bachhawat A.K., Yadav S. (2018). The glutathione cycle: Glutathione metabolism beyond the γ-glutamyl cycle. IUBMB Life.

[35] Xue Z., Nuerrula Y., Sitiwaerdi Y., Eli M. (2024). Nuclear factor erythroid 2-related factor 2 promotes radioresistance by regulating glutamate-cysteine ligase modifier subunit and its unique immunoinvasive pattern. Biomol. Biomed..

[36] Bao H., Chen Y., Meng Z., Chu Z. (2024). The causal relationship between CSF metabolites and GBM: A two-sample mendelian randomization analysis. BMC Cancer.

[37] Nirgude S., Choudhary B. (2021). Insights into the role of GPX3, a highly efficient plasma antioxidant, in cancer. Biochem. Pharmacol..

[38] Yoboue E.D., Rimessi A., Anelli T., Pinton P., Sitia R. (2017). Regulation of calcium fluxes by GPX8, a type-II transmembrane peroxidase enriched at the mitochondria-associated endoplasmic reticulum membrane. Antioxid. Redox Signal..

[39] Concetti J., Wilson C.L. (2018). NFKB1 and cancer: Friend or foe?. Cells.

[40] Asadi M., Taghizadeh S., Kaviani E., Vakili O., Taheri-Anganeh M., Tahamtan M., Savardashtaki A. (2022). Caspase-3: Structure, function, and biotechnological aspects. Biotechnol. Appl. Biochem..

[41] Petit P.X. (2025). Multiple entanglements of different cell death pathways, in which caspase-8 and BID interact with cardiolipin*, have been identified. Front. Cell Dev. Biol..

[42] Kayagaki N., Webster J.D., Newton K. (2024). Control of cell death in health and disease. Annu. Rev. Pathol..

[43] Barreyro F.J., Holod S., Finocchietto P.V., Camino A.M., Aquino J.B., Avagnina A., Carreras M.C., Poderoso J.J., Gores G.J. (2015). The pan-caspase inhibitor emricasan (IDN-6556) decreases liver injury and fibrosis in a murine model of non-alcoholic steatohepatitis. Liver Int..

[44] Zhang X., Nan H., Guo J., Liu J. (2021). NLRP12 reduces proliferation and inflammation of rheumatoid arthritis fibroblast-like synoviocytes by regulating the NF-κB and MAPK pathways. Eur. Cytokine Netw..

[45] Liu B., Zhu L., Bian L., Wen D., Cui X. (2025). Recent advances in the dual effects of activin a on tumors. Biochem. Biophys. Res. Commun..

[46] Bian X., Snow Z.K., Zinn C.J., Gowan C.C., Conley S.M., Bratulin A.L., Elhusseiny K.M., Miller J., Tchkonia T., Kirkland J.L. (2025). Activin A antagonism with follistatin reduces kidney fibrosis, injury, and cellular senescence-associated inflammation in murine diabetic kidney disease. Kidney360.

