# Supplementary material for: Integrated Liver Multi-Omics Reveals Beneficial Effects and Potential Mechanisms of Noni Fruit Flavonoids on Antioxidant Status and Anti-Inflammatory Responses in Cashmere Goats
Source: Antioxidants (Basel). 2026 Aug 9;15(8):987. doi: 10.3390/antiox15080987 (PMC13510141; doi:10.3390/antiox15080987)
Supplement: Supplementary file 1 [file antioxidants-15-00987-s001.zip › antioxidants-4390537-supplementary.pdf]

Supplementary Materials for Integrated Liver Multi-Omics Reveals the Beneficial Effects and Potential Mechanisms of Noni Fruit Flavonoids on Antioxidant Status and Anti-Inflammatory Responses in Cashmere Goats.

Table S1. Candidate gene primer sequences

**Genes**[1]	**Primer pairs(5′-3′)**	**GenBank number**	**Length（bp）**	**Annealing temperature（℃）**
GCLM	F: CAACTTGATGAGGAGTGACGC<br>R: ATCTGGGCTGATTTGGGAAC	XM_018045780.1	214	58
GPX8	F: CCATTCCACTTCAGCGTCTT<br>R: TTCCACCTTGGTTCCTTCTT	XM_005694704.3	203	58
GPX3	F: CCATTCGGTCTGGTCATTCTG<br>R: CCGTTCACATCGCCTTTCTC	XM_005683183.3	155	60
NFKB1	F: CTCCGAGCAAGTAACAGAAAC<br>R: GCATAGTCAAAGAGGGCATT	XM_018049264.1	180	55
ACVR1C	F: CGTAGTGACAGTGGCTGGAAT<br>R: CGATGAGCGATAGCAGGTTT	XM_005676114.3	111	58
IL1B	F:TGTCTTGCATTGCACTAACTCTTG<br>R: TTCTTTCATTGTGTTCCCCGTAG	NC_019460.2	161	55
NLRP12	F: GCCCATAGGGTCACAAAGAGT<br>R: GAAGTTGCGCCATGTAGTCC	XM_018063018.1	290	58
CASP3	F: ACCGAGGTTTAGTGCTGATG<br>R: CGTATGGACCACCGAGTTC	XM_018041755.1	184	55
PPARGC1A	F: GTACCAGCACGAAAGGCTCAA<br>R: ATCACACGGCGCTCTTCAA	NC_030813.1	183	57
PRKAA2	F: TTGATGATGAGGTGGTGGAG<br>R: CCGTGAGAGAGCCAGAGAGT	XM_018044652.1	138	57
YWHAZ	F: TGTAGGAGCCCGTAGGTCATCT<br>R:TTCTCTCTTATTCTCGAGCCATCT	AY_970970	102	60
β-actin	F：ACTGGGACGACATGGAGAAGA<br>R：GCGTACAGGGACAGCACAG	XM_018039831.1	199	60
B_2M	F: GGTGCTGCTTAGAGGTCTCG<br>R: ACGCTGAGTTCACTCCCAAC	NM_001009284	109	60

[1]*GCLM*= glutamate-cysteine ligase, modulatory subunit; *GPX*= glutathione peroxidase; *NFKB1*= nuclear factor kappa B subunit 1; *ACVR1C*= activin A receptor type 1C; *IL1B*=interleukin-1 beta; *NLRP12*= NOD-like receptor family pyrin domain containing 12; *CASP3*= caspase-3; *PPARGC1A*= peroxisome proliferator-activated receptor gamma coactivator 1-alpha; *PRKAA2*= protein kinase AMP-activated catalytic subunit alpha 2; *YWHAZ*= tyrosine 3-monooxygenase/tryptophan 5-monooxygenase activation protein zeta; *β-actin*= beta-actin; *B_2M*=beta-2-microglobulin
